# Vascularized versus Free Nerve Grafts: An Experimental Study on Rats

**DOI:** 10.3390/jpm13121682

**Published:** 2023-12-04

**Authors:** Giuseppe Giglia, Fernando Rosatti, Antonino Giulio Giannone, Giuditta Gambino, Maria Grazia Zizzo, Ada Maria Florena, Pierangelo Sardo, Francesca Toia

**Affiliations:** 1 Department of Biomedicine, Neuroscience and Advanced Diagnostics (Bi.N.D.), University of Palermo, 90127 Palermo, Italy; giuseppe.giglia@unipa.it (G.G.); giuditta.gambino@unipa.it (G.G.); pierangelo.sardo@unipa.it (P.S.); 2 Euro-Mediterranean Institute of Science and Technology (IEMEST), 90139 Palermo, Italy; 3 Plastic and Reconstructive Surgery, Department of Surgical, Oncological and Oral Sciences, University of Palermo, 90127 Palermo, Italy; fernando.rosatti@unipa.it; 4 Department of Health Promotion Sciences, Maternal and Infant Care, Internal Medicine and Medical Specialties (PROMISE), University of Palermo, 90127 Palermo, Italy; giulio.giannone@unipa.it (A.G.G.); adamaria.florena@unipa.it (A.M.F.); 5 Department of Biological, Chemical and Pharmaceutical Sciences and Technologies (STEBICEF), University of Palermo, Viale delle Scienze, 90127 Palermo, Italy; mariagrazia.zizzo@unipa.it; 6 ATeN (Advanced Technologies Network) Center, University of Palermo, Viale delle Scienze, 90127 Palermo, Italy

**Keywords:** vascularized nerve grafts, nerve grafts, nerve regeneration, nerve animal models, peripheral nerves

## Abstract

Background: Vascularized nerve grafts (VNGs) have been proposed as a superior alternative to free nerve grafts (FNGs) for complex nerve defects. A greater regenerative potential has been suggested by clinical and experimental studies, but conclusive evidence is still lacking. Methods: In this experimental study, 10 adult male Wistar rats received a non-vascularized orthotopic sciatic nerve graft on their right side, and a vascularized orthotopic sciatic nerve graft nerve on their left side. Functional outcome following nerve regeneration was evaluated through electrodiagnostic studies, target muscles weight and histomorphology, and data of VNGs and FNGs were compared. Results: The results of this study showed a significant difference in the motor unit number of Gastrocnemius Medialis (GM) estimated by MUNE in the VNG side compared to the FNG side. No other significant differences in axonal regeneration and muscle reinnervation were evident at either electrodiagnostic, histomorphology studies or muscle weight. Conclusions: This experimental model showed slight differences in nerve regeneration between VNGs and FNGs, but cannot support a high clinical advantage for VNGs. The results of this study show that VNGs are not strongly superior to FNGs in the rat model, even in avascular beds. Clinical advantages of VNGs are likely to be limited to extensive and thick nerve defects and can only be assessed on experimental model with bigger animals. Also, we showed that the MUNE technique provided a reliable and reproducible evaluation of functional outcomes in the rat sciatic nerve and defined a reproducible protocol for functional evaluation of muscle reinnervation.

## 1. Background

Vascularized nerve grafts (VNGs) have been proposed as a superior alternative to free nerve grafts (FNGs) for complex nerve defects. However, current gold standard in treating a nerve gap is an autologous FNG and indications for VNGs are limited due to their technical difficulty, their cost-to-benefit ratio, and a lack of clear evidence for their superiority [1,2,3,4]. In particular, indications include very long (more than 6 cm) or large caliber nerve defects, scarred, traumatized, irradiated and/or poorly vascularized recipient bed, old patients, and patients with long duration of denervation [5,6,7,8,9].

A greater regenerative potential has been suggested by clinical and experimental studies, but conclusive evidence is still lacking. Also, harvesting of VNGs yields a significant morbidity that needs to be balanced with the degree of clinical improvement they can bring. Their clinical use is limited by several factors, morbidity of the donor site related to nerve harvesting is one of the most important: some nerves—for example the ulnar, median and tibial nerve—have a reliable vascularization suitable for their microsurgical harvesting on a single vascular pedicle, but have no clinical use due to the severe functional deficit that would ensue from their removal. Morbidity of the donor site related to the removal of the vessels in another important factor to be considered: the removal of other nerves, such as the sensory radial and the deep peroneal (distal portion), while entailing an acceptable functional morbidity limited to a sensory deficit requires the sacrifice of important vascular axes (radial artery and anterior tibial artery, respectively), which limit their clinical applications [10]. Variability of vascular anatomy also needs to be taken into account [11]. The sural nerve is by far the most used among the VNGs as it owns several favorable characteristics [12,13,14,15]. Its anatomy is constant: it arises from the union of the medial and lateral cutaneous nerve of the calf or from the tibial and common peroneal; also, it can be removed without sacrificing a main vascular axis and with an acceptable functional morbidity.

Although recovery after a nerve injury is not always satisfactory, clinical research must provide answers to the over 5 million cases of peripheral nerve injury that occur worldwide every year. Some authors believe that progress in the field of nerve lesions is linked to the application of tissue engineering principles and materials, rather than to the development of cutting-edge microsurgical instruments and techniques. Although some experimental results are promising, to date, autografts still remain the first reconstructive choice in this type of trauma [1].

A recent systematic review from our group suggests better early but similar long-term results for VNGs compared to FNG [16]. However, the existing literature is heterogenous and not conclusive, and no standard animal model or evaluation method has been validated.

The aim of this experimental study was to compare functional outcomes of non-vascularized vs. vascularized nerve graft in an experimental rat model. The animal model and the evaluation methods were designed to address the evidence and the shortcomings of the literature, and to specifically respond to two questions:

Is the rat model appropriate to compare the outcomes of VNGs to FNGs? If any significant difference between VNGs and FNGs exists, is it only statistically or also clinically significant?

## 2. Materials and Methods

The study received prior approval from the Italian Ministry of Health, (n 270/2016-PR del 11 March 2016) and experiments were conducted according to Italian and European legislation.

Power analysis determined that 9 animals per group provide 80% statistical power with an alpha of 0.05. This calculation is based on an expected 20% effect on the motor unit number between the two nerve graft types. This threshold was based both on evidence from literature data and clinical translational potential of the study results, as differences < 20% are not likely to be “clinically” significant [5,17].

The experimental group included ten adult male Wistar rats (weight 350–400 g) which received a non-vascularized orthotopic sciatic nerve graft on their right side, and a vascularized orthotopic sciatic nerve graft nerve on their left side.

### 2.1. Surgical Procedure:

Animals (N = 10) were anesthetized with Xylazine (10 mg/kg) e Ketamine (100 mg/kg). They received antibiotic prophylaxis with enrofloxacin (2.5 mg/kg/die for 7 days) and antalgic prophylaxis with meloxicam (0.3 mg/kg/die).

The sciatic nerve was exposed bilaterally through an anterior approach in the inguinofemoral region, from its emergence beneath the piriformis muscle to its bifurcation into the tibialis and peroneus communis.

Before nerve section and nerve graft, electrophysiologic studies were performed (see below).

In the right side, the nerve was skeletonized, dissecting out its vascular supply and interrupting the ascending branch to the nerve of the caudal femoral artery. A 15 mm graft was then harvested and orthotopically sutured to the proximal and distal stumps of the sciatic nerve with 2 interrupted 10/0 nylon sutures. The graft was enveloped in a 0.12 mm thick silicon sheet (Folioxane, Novatech, France, La Ciotat) to prevent revascularization from surrounding tissues. The skin was then closed with interrupted 3/0 silk sutures.

In the left side, the nerve was isolated, respecting its perineural vessels and the ascending branch to the nerve of the caudal femoral artery. A 15 mm graft was then harvested—pedicled on the ascending branch to the nerve of the caudal femoral artery—and orthotopically sutured to the proximal and distal stumps of the sciatic nerve with 2 interrupted 10/0 nylon sutures. The graft was enveloped in a 0.12 mm thick silicon sheet (Folioxane, Novatech, France, La Ciotat) to prevent revascularization from surrounding tissues. The skin was then closed with interrupted 3/0 silk sutures.

### 2.2. Evaluation

Functional outcome following nerve regeneration was evaluated through electrodiagnostic studies, targeting muscle weight and histomorphology, and data of VNGs and FNGs were compared.

Electrodiagnostic studies: estimation of the motor unit number with the MUNE technique for the tibialis anterior muscle (from the peroneus communis nerve) and for the gastrocnemius muscle (from the tibialis nerve) was performed at baseline (T0: after nerve exposure but before surgery on the nerve), 6 weeks after surgery (T1: under general anesthesia but through percutaneous stimulation) and at 12 weeks (T2: after nerve exposure and before animal sacrifice) according to an adapted version of the protocol by Gordon et al. [18,19,20,21,22].

The animal was briefly anesthetized as previously described, and held in supine position, with the lower limbs gently spread, over a thermoregulated heating pad, in order to keep a constant temperature of 37°.

A two-needle bipolar stimulating electrode (inter-needle distance 5 mm) was then positioned on the sciatic nerve in the inguinofemoral region, the optimal position for stimulation was found and kept constant using a mechanical device.

CMAP was then evoked by stimulating the nerve at supramaximal intensity (i.e., 10% above threshold) with a square biphasic stimulus, duration of 0.01 ms and recorded through a pair of monopolar needle electrodes, in a belly tendon montage over the Tibialis Anterior (TA) or Gastrocnemius Medialis (GM) muscles. Then, stimulation intensity was reduced to 0 and gradually increased in steps of 1 mA, at random time intervals, until a reproducible, “all-or-none” abrupt increase in evoked response was recorded (Single Motor Unit Action Potential- sMUAP). Fifteen SMUAPs were recorded for each muscle.

Motor Unit Number was estimated by the formula:

Peak-to-peak amplitude of the maximum CMAP/Peak-to-peak amplitude of the average S-MUAP

### 2.3. Muscle Weight

After animal sacrifice (12 weeks), the wet muscle of the right and left anterior tibial muscles and gastrocnemius muscles were recorded.

### 2.4. Histomorphology

**Figure 1:**
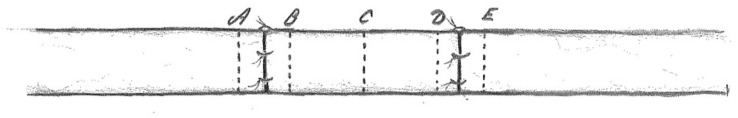
Sections of the sciatic nerve were obtained 2 mm proximal (A) and 2 mm distal (B) to the proximal nerve suture, in the middle part of the nerve graft (C), 2 mm proximal (D) and 2 mm distal (E) to the distal nerve suture.

After nerve sacrifice (12 weeks), sections of the sciatic nerve were obtained bilaterally:

- 2 mm proximal and 2 mm distal to the proximal nerve suture;
- in the middle part of the nerve graft;
- 2 mm proximal and 2 mm distal to the distal nerve suture (Figure 1).

Hematoxylin and Eosin, Toluidine Blue, and Masson Trichrome staining was performed. Moreover, each section was stained with immunohistochemical staining for the endothelial vascular marker ERG, that highlights with a brown chromogen the nuclei of the vascular endothelial cells. ERG immunohistochemistry was performed with the rabbit monoclonal antibody EP111 (Cell Marque), using the Leica Bond III autostainer, and the following data were recorded for each of the 5 groups of sections.

- transverse nerve diameter;
- diameter and density of myelinated axons (axons/mm^2^);
- diameter and density of nerve fibers (fibers/mm^2^);
- neural/connective tissue area rate;
- number of axons/nerve fibers rate.
- microvascular density of the nerve.

### 2.5. Statistical Analysis

Statistical analysis was performed using “Statistica software” (version 8.0; Dell Software, Tulsa, OK, USA, www.statsoft.com (accessed on 15 January 2023)). 

One rat was excluded from analysis as at the time, 1 presented a self-inflicted amputation of the right hindlimb. 

Electrophysiological data at time 0, 1 and 2, and histomorphology data from the different section level were analyzed and compared for each sciatic nerve; electrophysiological, and histomorphology data from the right and the left sciatic nerves were analyzed and compared through repeated measure ANOVA. Bonferroni post hoc test was applied when appropriate. Muscle weights were compared by means of a *t*-test.

## 3. Results

### 3.1. Electrodiagnostic Studies

**Figure 2:**
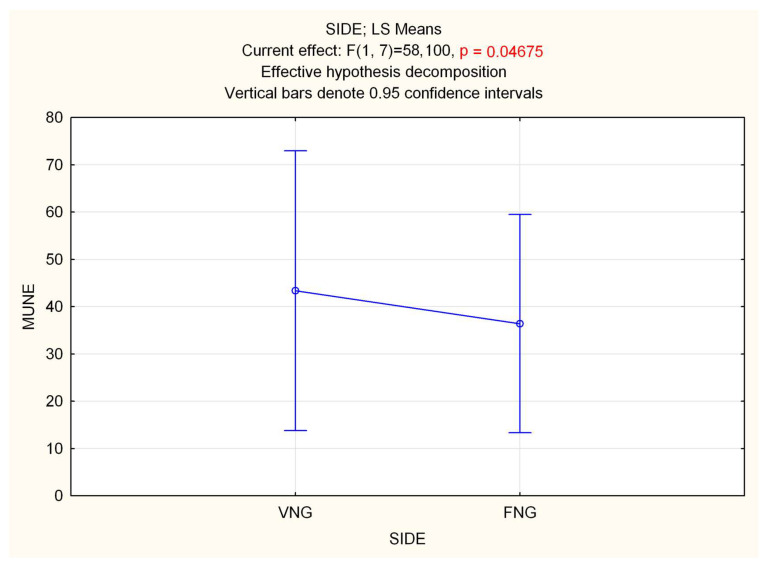
Statistical analysis of MUNE difference performed on side (VNGs vs. FNGs) in the GM muscle, showing a slight but significant decrease in MUNE value for FNGs side.

Repeated measure (Rm) ANOVA on MUNEs showed a significant effect of time in both GM (F 2.16 = 27.996, *p* < 0.0001) and TA (F 2.16 = 130.42, *p* < 0.0001) muscles, with a decrease in MUNE. Statistical analysis performed on side (VNGs vs. FNGs), showed a significant difference in GM muscle, with a slight decrease in MUNE value for FNGs side (F 1.8 = 5.7188, *p* = 0.04) (Figure 2), while no differences emerged in TA (*p* = 0.8). RmANOVA on interaction side (VNGs vs. FNGs) × time (T0, T1, T2) revealed a significant main effect for GM muscle (F 2.16 = 4.4268, *p* < 0.03), which, however, was not due a difference of MUNE value over time between VNGs vs. FNGs muscle, as evidenced by Bonferroni post hoc analysis. No other significance was found.

### 3.2. Muscle Weight

There was a minimal difference in the wet muscle of the right and left anterior tibial muscles (0.5 vs. 0.4 g) and gastrocnemius muscles (1.2 vs. 1.1 g), which was not statistically significant.

### 3.3. Histomorphology

Mean values of the nerve diameter, axon diameter and density, fiber diameter and density, neural to connective tissue ratio and axons to fibers ratio are presented in Table 1.

**Figure 3:**
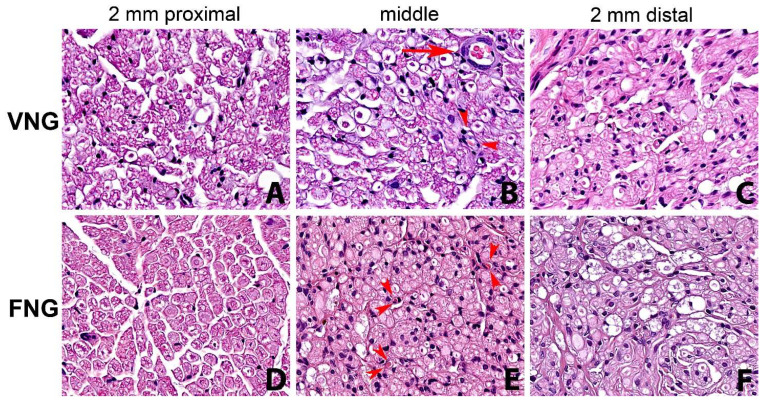
Series of nerve sections with hematoxylin-eosin staining (original magnification ×400). From left to right: 2 mm proximal, middle part of the nerve graft and 2 mm distal to the distal suture. (**A**–**C**) right side, (vascularized nerve graft); (**D**–**F**) left side (free nerve graft). Reduction in number of fibers and fiber degenerative and regenerative changes from proximal to distal are evident in both the vascularized nerve graft side and non-vascularized nerve graft, being more evident in the latter. Small arterioles (arrow) and capillaries (arrowheads) are observed in the right side (**B**). Small capillaries (arrowheads) are evident also in the left non-vascularized side (**E**). VNG: Vascularized nerve graft; AVG: Free nerve graft.

There was a statistically significant drop in the value of all measured parameters of the distal nerve section (n 5) compared to the proximal section (n 1). In particular, statistical significance emerged in nerve diameter: F(4.32) = 2.8881, *p* = 0.0378; axon HPF: F(4.32) = 17.913, *p* = 0.0000; fiber HPF: F(4.32) = 8.6465, *p* = 0.0000 and axons/fibers: F(4.32) = 8.8093, *p* = 0.0000. All considered parameters were slightly higher in the left (VNG) side. Fiber degenerative phenomena from proximal to distal were more evident and connective tissue was more represented in the non-vascularized (FNGs) nerve graft side (Figure 3).

**Figure 4:**
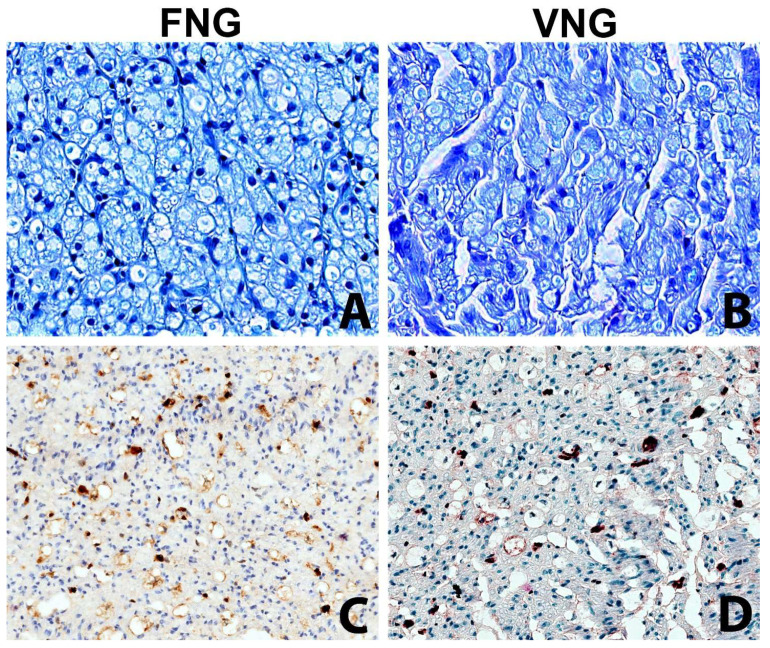
The intraneural microvascular density was assessed with the immunochemistry in VNG and FNG animal’s nerve sections (**A**,**B**) Nerve section of the nerve grafts 2 mm distal to the distal suture stained with Toluidine Blue (original magnification ×400). (**A**) left side, free nerve graft. (**B**) right side, vascularized nerve graft. No substantial differences between the two conditions. (**C**,**D**) Nerve section of the nerve grafts 2 mm distal to the distal suture stained with vascular endothelial marker (ERG) to assess the microvasculature (original magnification ×200); (**C**) left side, free nerve graft. (**D**) right side, vascularized nerve graft. The immunohistochemical stain highlights with a brown chromogen the nuclei of endothelial cells of intraneural microvessels, showing the presence of small capillaries in both sides.

The intraneural microvascular density assessed with immunohistochemistry for the endothelial marker ERG showed no significant differences between the vascularized and non-vascularized side (Figure 4).

## 4. Discussion

The treatment of nerve lesions has always been a clinical challenge, with often unsatisfactory results. In situations where a tensionless suture of the two ends of the nerve is not possible, the gold standard is an autologous non-vascularized nerve graft [2]. The treatment of these lesions using a nerve graft is always inferior in terms of outcome for the patient when compared to primary coaptation, due to the presence of an anastomosis on two sides that increases the surface that must regenerate, the ischemia of the graft and often a bed poor vascularity [23].

VNGs are technically far more difficult to harvest compared to FNG. Also, they yield a higher morbidity. Thus, their clinical use needs to be justified by a significantly superior result [24]. The results of this study showed a significant difference in the motor unit number of GM estimated by MUNE in the VNG side compared to the FNG side; no other significant differences in axonal regeneration and muscle reinnervation were evident at either electrodiagnostic, histomorphology studies, or muscle weight. This experimental model showed slight differences in nerve regeneration between VNGs and FNGs, but cannot support a high clinical advantage for VNGs. Also, we applied the MUNE technique to the rat sciatic nerve and defined a reproducible protocol for functional evaluation of muscle reinnervation.

Recently, we performed a systematic review on an experimental model of VNGs [16], based on which we designed our experimental model and evaluation methods. The rat model is by far the most commonly used animal model for the study of nerve regeneration, including the study of VNGs, despite only short nerve gaps being obtained (<3 cm). For this reason, we chose to use the rat as the experimental model, but taking into account the main limitations of the previously published models. Also, we decided to focus on electrophysiology and histomorphology as some papers reported poor correlation of sciatic functional index (SFI) with other measures of reinnervation [25]. 

The literature on the experimental evidence of a superiority of VNGs over FNGs is controversial and results vary significantly on the basis of the animal model, length of the nerve grafts and vascularization of the receiving bed [2]. Some authors support a better axonal regeneration, remyelination and functional recovery, especially in avascular beds [5,19,26]. Others, however, failed to demonstrate a significant difference in their long-term results [27,28,29].

Actual clinical indications for VNGs are limited to long nerve defects and/or avascular receiving beds. Despite that, most previous studies were performed on rats with short nerve defects and vascular beds; also, different and incomplete evaluation methods were often used. For this reason, we designed a rat model on an avascular bed with multiple standardized evaluation methods (both electrodiagnostic and histomorphologic) to focus on comparison of VNGs and FNGs.

Also, we refined the MUNE technique [22,30], a well-established clinical method, for evaluation of muscle reinnervation, for experimental use in the rat hindlimb.

Baseline values were obtained at time 0 at the beginning of surgery following nerve exposure and at 12 weeks before animal sacrifice. Preliminary experiments (data not shown) showed that percutaneous stimulation provided comparable MUNE results and was then used for the intermediate 6 weeks post-operative evaluation. According to the literature, estimation of the motor unit number with the MUNE technique provided a reliable index of muscle reinnervation, also showing the expected reduction of MUNE over time [30]. Electrophysiological findings showed an apparently better reinnervation in GM muscle in VNG condition as compared to FNG, but not in the TA. This evidence could allow speculation that tibialis nerve fibers can take more advantage from vascularized graft rather than peroneal fibers, which are known to be more vulnerable to injuries [31]. If confirmed in a larger series of experiments, this outcome could have a translational impact on the surgical approach to tibialis nerve injuries in human patients. On the other end, although statistically significant, the difference in the estimated motor unit number was only slight, which could suggest a limited clinical advantage for VNGs, also considering that the sample size was specifically calculated based on the motor unit number as primary endpoint and the study sample was slightly superior to that estimated for statistical significance.

The difference in GM vs. TA muscle reinnervation was recorded at both 6 and 12 weeks; however, besides being the only significant difference found in this study, a decrease of this difference over time cannot be fully excluded, as previous studies with a longer follow-up showed better early but similar long-term results for VNGs compared to FNGs [27,28,29]. This study also strengthens a base of knowledge supporting MUNE as an easy to reproduce, consistent and valuable approach for evaluating neuromuscular function in rodent nerve repair injury models [22].

Also, no statistically significant difference was found in the wet muscle of right and left anterior tibial muscles and gastrocnemius muscles.

Histomorphology evaluation included a standardized study of nerve diameter, axon diameter and density, fiber diameter and density, neural to connective tissue ratio and axons to fibers ratio of the nerve graft and both proximal and distal to it. Statistical analysis of this data did not show a significant difference in any of these parameters, despite a slightly better preservation of blood supply, less fibrosis and less degenerative phenomena in the VNGs, but only a significant drop in the value of all measured parameters of the distal nerve section (n 5) compared to the proximal section (n 1).

Overall, the results of this study suggest that the clinical advantage of VNGs over FNGs is limited, at least for short nerve gaps.

Considering that the study sample was superior to the estimated statistically significant sample and that the evaluation methods were standardized and optimized based on a literature review, the results of this study also question the appropriateness of the rat model for the study of vascularized nerve grafts.

The only slight superiority of VNGs over FNGs shown by this study does not support the complexity of this clinical procedure for short nerve defects even in avascular beds, and suggests that the time and resources required to repair a large traumatic nerve gap are better spent on obtaining a nerve scaffold in a timely manner, regardless of vascular status.

Based on the results of this study, we also suggest that the short nerve gaps (<2–3 cm) that can be created in the rat models are not suitable to evaluate the superiority of VNGs and FNGs for clinical use, and only studies on bigger animals and larger and thicker nerve gaps could address this question [32].

The real advantage of VNGs over FNGs is difficult to be evaluated in clinical series, as complex nerve defects are very heterogeneous, making results difficult to compare and controlled clinical trials difficult to be designed. Experimental studies could better fit this purpose. Most published experimental studies on VNGs were performed on rats, which, despite being the reference model for nerve regeneration, do not appear to be the best model for the particular purpose of studying VNGs.

In the clinical field today, the use of vascularized nerve grafts still plays a fundamental role [8]; however, as previously mentioned, this type of procedure involves morbidity at the donor site which can sometimes be poorly tolerated. This has meant that clinical research in tissue engineering has been directed in recent years towards seeking less invasive and equally or more performing responses from a regenerative point of view [32].

In the context of clinical research, many different techniques and materials have been proposed (mainly PVC and silicone) to reproduce biological vascularized conduits for the reconstruction of nerve gaps. The procedure involves the insertion of a conduit into a vascular bed or next to a donor nerve for a period of time ranging from 1 to three weeks; subsequently, the inserted duct will be resurfaced by a newly formed pseudo synovial sheet, a biological membrane that can be reused as a bridge between two nerve stumps in the reconstruction of small defects [10].

Another important role in the future perspectives is represented by Engineered Neural Tissues. A study published by Gao et al. in 2013 demonstrated that by simultaneously culturing Schwann cells and endothelial cells within fiber-reinforced 3D composite scaffolds, vascularized nerve constructs could be obtained. By testing them on the sciatic nerve of murine models, the researchers demonstrated how these constructs were useful in promoting nerve repair in terms of myelin thickness, conduction speed, and number of nerve fibers [33].

Although these techniques are very promising as they would allow for nerve reconstruction without the morbidity of the donor site, further large-scale preclinical and clinical studies are useful for validation and use in clinical practice.

A limitation of this study is that the vascularized nerve grafts were not compared to a healthy nerve or simple repair. However, this control group was omitted in respect of the 3R principles, as we already know from the literature that nerve reconstruction with a graft is inferior to simple nerve repair, and the aim of the study was to compare the standard technique for reconstruction of a nerve gap (FNG) to a potential superior option (VNG).

Another limitation of the study is that results were evaluated by electrodiagnostic studies and histomorphology, but not supported by gait or locomotion behavioral assessments. The Sciatic Functional Index (SFI) value, a well-established and commonly used method for assessment of motor nerve recovery after sciatic nerve injury, was not calculated, as other assessment methods were chosen. However, calculating the SFI could have been useful for comparison with other studies. Also, histomorphology was assessed by an optical microscope while transmission electron microscopy could have been more accurate.

## 5. Conclusions

The results of this study show that VNGs are not strongly superior to FNGs in the rat model, even in avascular beds. Clinical advantages of VNGs are likely to be limited to extensive and thick nerve defects and can only be assessed on experimental model with bigger animals.

Also, we showed that the MUNE technique provided a reliable and reproducible evaluation of functional outcomes in the rat sciatic nerve and defined a reproducible protocol for functional evaluation of muscle reinnervation.

Although vascularized and free nerve grafts still represent the most advantageous choice in clinical practices to date, research in the field of tissue engineering could represent the future, obtaining similar results while reducing morbidity at the donor site.

## Figures and Tables

**Table 1:** Detailed data on histomorphologic nerve characteristics. Mean values. HPF: high-power field.

Side	Level	Nerve Diameter (mm)	Axon Diameter (μm)	Axons/5 HPF	Fiber Diameter (μm)	Fiber/5 HPF	Neural/Connective Tissue	Axons/Fibers
Right	1	1.01	58.33	2.53	8.43	84.33	3.33	0.59
	2	1.45	61.67	3.27	8.83	105.33	3.33	0.61
	3	1.21	45.67	2.67	8.03	90.33	3	0.54
	4	1.13	28	2.07	7.53	57.33	2.67	0.49
	5	0.89	27.67	3	8.67	49.67	3	0.52
Left	1	1.16	34	2.4	6.97	64.67	2.33	0.48
	2	0.79	35	3.47	8.73	79.33	2	0.49
	3	0.84	27.67	2.7	6.9	55.33	2.33	0.58
	4	0.79	42.33	2.63	8.07	78.67	2.67	0.54
	5	0.65	19.33	2.1	7.27	40.67	2	0.47
		*p* = 0.0378		*p* = 0.0000		*p* = 8.6465		*p* = 0.0000

## Data Availability

All data generated or analyzed during this study are included in this published article.

## List of Abbreviations

VNG	Vascularized nerve grafts
FNG	Free nerve grafts
TA	Tibialis Anterior
GM	Gastrocnemius Medialis
SFI	Sciatic Functional Index
